# scFASTCORMICS: A Contextualization Algorithm to Reconstruct Metabolic Multi-Cell Population Models from Single-Cell RNAseq Data

**DOI:** 10.3390/metabo12121211

**Published:** 2022-12-02

**Authors:** Maria Pires Pacheco, Jimmy Ji, Tessy Prohaska, María Moscardó García, Thomas Sauter

**Affiliations:** 1 Department of Life Sciences and Medicine, University of Luxembourg, 4367 Belvaux, Luxembourg; 2 Luxembourg Centre for Systems Biomedicine, University of Luxembourg, 4367 Belvaux, Luxembourg

**Keywords:** single-cell RNAseq, metabolism, metabolic modelling, algorithm

## Abstract

Tumours are composed of various cancer cell populations with different mutation profiles, phenotypes and metabolism that cause them to react to drugs in diverse manners. Increasing the resolution of metabolic models based on single-cell expression data will provide deeper insight into such metabolic differences and improve the predictive power of the models. scFASTCORMICS is a network contextualization algorithm that builds multi-cell population genome-scale models from single-cell RNAseq data. The models contain a subnetwork for each cell population in a tumour, allowing to capture metabolic variations between these clusters. The subnetworks are connected by a union compartment that permits to simulate metabolite exchanges between cell populations in the microenvironment. scFASTCORMICS uses Pareto optimization to simultaneously maximise the compactness, completeness and specificity of the reconstructed metabolic models. scFASTCORMICS is implemented in MATLAB and requires the installation of the COBRA toolbox, rFASTCORMICS and the IBM CPLEX solver.

## 1. Introduction

Single-cell analysis has the potential to significantly improve our understanding of complex biological systems, with cancer research being one major application among many others. Tumours are composed of heterogeneous populations of cells that can carry various oncogenic mutations and are often subjected to a substantially different environment. These factors can significantly alter their metabolism [1,2]. Depending on the availability of nutrients and oxygen, some cancer cell populations favour glycolysis as the principal energy production pathway, whilst other cancer cells might uptake lactate released from adjacent stromal cells in the tumour microenvironment (TME) to fuel oxidative phosphorylation (OXPHOS) pathways [3]. The different metabolisms of cancer cell populations let them respond differently to treatments. Eventually, the patient might become resistant to the treatment due to non-responsive cell populations [4,5].

Understanding the metabolism of each cancer cell population and their interaction with the TME will shed light on the metabolic mechanism sustaining cancer progression and resistance. The TME is scarce in nutrients, and hence, cells within the TME compete for resources. However, cancer cell populations can also collaborate by secreting pro-angiogenesis or survival signals to favour proliferation, and migration [6]. Furthermore, cancer cells can change the TME, notably through acidosis and the accumulation of lactate or the depletion of nutrients, which allows them to escape the immune system. T cells have to undergo a metabolic rewiring from OXPHOS to glycolysis to sustain biosynthesis pathways and acquire their effector function [7]. However, the adverse conditions in the TME, notably acidosis, prevent glycolysis and OXPHOS in T cells [8]. In addition, cancer cells can induce the reprogramming of other cell types such as cancer-activated fibroblasts (CAFs) and adipocytes to obtain lactate, amino acids, lipids, and Reactive Oxygen Species (ROS) [9].

Single-cell RNAseq data, which capture the tumour heterogeneity by counting the reads for every individual cell, allow clustering cancer cells according to their expression pattern, thereby unravelling the role of the different populations. So far, bulk RNAseq-based context-specific metabolic models have been successfully applied to simulate exchanges between cancer cells and the gut microbiome [10,11] or to predict repurposable drugs [12], amongst many other applications. However, metabolic models reconstructed from single-cell RNAseq data could describe the metabolism of each cell population more accurately and with higher resolution compared to bulk RNAseq-based context-specific metabolic models. A single-cell-based metabolic model [13] was published in 2019 that comprises 271 reactions of the core metabolism (coreHMR, core metabolism extracted from the HMR model [14]) but not of the full genome scale. Rohlenova et al. [15] did use the larger Recon1 model as generic reconstruction input [16]. However, the context-specific model was reconstructed with GIMME [17] and bulk RNAseq data. The single-cell RNAseq data was only used to eliminate reactions associated with genes that were not expressed, thus not making full use of the information provided by single-cell data. Other related works aimed at refined Flux Balance Analysis (FBA) rather than building a metabolic model. As an example, SSA-FBA [18] used a stochastic model to estimate gene expression and used the estimates as input for FBA instead of integrating single-cell data, whereas Compass [19], an FBA algorithm that uses single-cell transcriptomic profiles while optimising for an objective function to penalise the reactions associated with low expression in the network. Finally, Alghamdi et al. [20] retrieved metabolic maps from the KEGG database [21] which were reduced, curated, and converted into a directed factor graph. The resulting map contained 1880 reactions. Single-cell data was then mapped onto the network and a left truncated Gaussian curve was fitted to the expression data to identify expressed genes. Modules were excluded from the analysis if they disconnected from the network due to the presence of non-expressed genes at key positions. Finally, incorporating the topological and expression data, the workflow aims to maximise consistency between the gene expression and the predicted flux. In all, to our knowledge, there is no context-specific metabolic model reconstruction tool allowing the use of single-cell data at the genome-scale level.

In this paper, we propose scFASTCORMICS, an algorithm to reconstruct context-specific multi-cell population genome-scale metabolic models (multi-cell population model) from single-cell RNAseq data. This reconstruction allows simulating the metabolism within the different cell clusters and also the exchanges between the clusters and the TME. scFASTCORMICS and the underlying rFASTCORMICS can be downloaded from https://github.com/sysbiolux/scFASTCORMICS accessed on 21 September 2022.

## 2. Experimental Design

**Figure 1:**
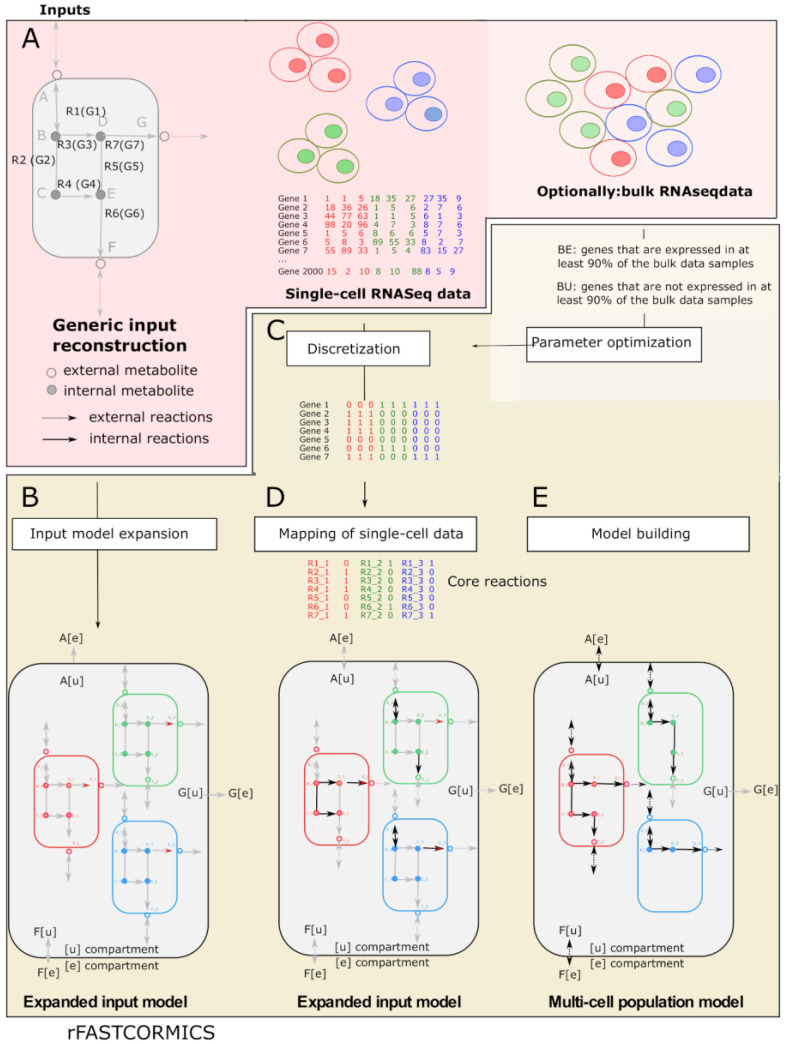
Workflow: The inputs of scFASTCORMICS are a generic model; in the paper, Recon3D, (gray box in panel (**A**), metabolites and reactions are represented in circles and arrows, respectively) single-cell data and optionally medium constraints and bulk RNAseq data from the same context. Generic reconstructions or models contain every metabolic reaction described in an organism regardless of the context or cell type. The generic model has internal reactions (not connected with the environment, dotted arrows) and external reactions (connected with the environment, solid arrows). To create an expanded input model (large grey box in panel (**B**–**D**)) that accounts for the dataset’s different populations (colored boxes in the large grey box), a set of internal reactions is created for each population by duplicating the internal reactions of the generic model. The exchange reactions between the system and the environment in Recon3D are transformed into transporter reactions to the microenvironment in the expanded input model. The reversibility of the reactions is conserved. In other words, the sub-models corresponding to the cell populations can import and export the same metabolites as Recon3D. The external metabolites [e] (transparent circle) of Recon3D are converted into [u] metabolites ([u] standing for microenvironment). A set of reversible transporters and exchange reactions are added to the model to transport these metabolites between the microenvironment and the external compartment and to allow them to enter and exit the system. The microenvironment allows metabolite exchanges between the population sub-models (red, green, and blue boxes). The expanded input model is a generic model as the reactions included in the sub-models are identical. Medium constraints (inputs can be closed) and uptake rates can be applied to the bounds of the transporter reactions between the microenvironment and the sub-models for measured metabolites, or, if the data does not specify the population, the exchange reactions of the expanded input model can be constrained. Constraining models with media information allows to exclude or force reactions to be present in the multi-cell population model. The multi-cell population model is a consistent network corresponding to the active reactions and pathways in each population and is extracted from the expanded input model. Therefore, the genes in the single-cell data are discretized in expressed genes and genes with unknown expression status and mapped to the reactions to obtain a set of core (supported) and non-core (not supported) reactions. If bulk RNAseq data of the same context is available, an additional optimization can be run to obtain an optimal parameter setting for the dataset or the default parameter can be used for the discretization. FASTCORE [22] is then run on the core set and will add a close-to-minimal number of reactions required to obtain a consistent network (connect the core reactions) (**E**). The added non-core reactions can be internal reactions but also transporters to the microenvironment or exchange reactions if the path corresponding to the additions of the lowest numbers of reactions requires an exchange of metabolites between sub-models.

scFASTCORMICS is the latest member of the FASTCORE family [12,22,23,24], a family of algorithms that allows reconstructing context-specific metabolic models from a generic reconstruction by extracting a flux-consistent subnetwork that corresponds to the active pathways in a cell type or context of interest. In a consistent model, every reaction can carry a non-zero flux. In other words, the model does not contain gaps or dead ends that produce a metabolite without consuming it, or vice-versa. scFASTCORMICS, like the other members of the FASTCORE family, does not alter the bounds of the reactions in the generic reconstruction according to the expression level in the omics data. Instead, it uses a core expansion strategy in which a minimal set of reactions, with no or low evidence, is added to a core set of reactions with high evidence according to the omics data to obtain a compact flux consistent network (Figure 1). scFASTCORMICS is, to our knowledge, the first context-specific building algorithm using single-cell RNAseq data as input.

The use of single-cell data required the development of a new discretization workflow (run within scFASTCORMICS) to address the high drop-out rate characterizing single-cell RNAseq data and the building of an expanded network that contains a set of internal reactions for each cell population. The expanded input model is used to map the single-cell RNAseq data and extract a multi-cell population model. For an introduction to the underlying constraint-based modelling approach and the main computational tools see [25,26,27].

scFASTCORMICS implements as inputs single-cell RNAseq data pre-processed with the Seurat R package [28] or any other tool that allows assigning the cells to different clusters and a consistent generic metabolic reconstruction (Figure 1A). Here, Recon3D [29] was chosen, but the workflow is compatible with any model with a COBRA structure [25]. If the model contains blocked reactions, FASTCC [22] is run before scFASTCORMICS to obtain a consistent input network. Optionally, bulk RNAseq from the same context can be used as input to obtain the optimal parameter settings for the discretization of the respective single-cell RNAseq data.

Within scFASTCORMICS, an expanded input model is created from a generic reconstruction that includes subnetworks corresponding to each of the different clusters connected via the union compartment [u], representing the TME. Therefore, internal metabolites, reactions and associated genes of the input generic reconstruction are duplicated to obtain a set of internal metabolites, reactions and genes per cluster. The external metabolites are converted into [u] metabolites. The metabolites of this new compartment are further connected to the external compartment by exchange reactions. To mark the belonging of a metabolite, gene, or reaction to a cluster, a tag with the number of the cluster (_1, _2, etc.) is added to the metabolite’s identifiers (Figure 1B).

The critical step of the scFASTCORMICS workflow is the determination of the core reaction set, which requires a binarization of the single-cell data into expressed genes (“1”) and genes with unknown expression status (“0”) in a cluster. Hence, only those reactions regulated by expressed genes will be considered to be part of the core reaction set. As single-cell RNAseq data is characterised by a high level of noise and drop-outs [30], the absence of measured expression does not imply non-expression and hence reactions under the control of these genes are not necessarily inactive. Thus, two parameters are taken into consideration to determine if a gene is expressed in a cluster: (1) the Ranked Expression Intensity (REI) representing the expression level of a specific gene in a cell, ranked, vs. the expression levels of all genes in all cells and (2) the coverage standing for fraction of cells in a cluster that expressed this gene. Based on these discretization parameters, the sets of core reactions for every cluster are determined and mapped to the expanded input model (Figure 1C,D). FASTCORE is then run, generating a compact and consistent network model containing sub-models for each cluster, integrated by the union compartment representing the TME (Figure 1E).

Multi-cell population models can be employed as input models for analysis commonly used in metabolic modelling such as Flux Variability Analysis (FVA), Flux balance analysis (FBA), single or multiple knockouts, etc. In the present paper, the multi-cell population models were used for pathway analysis. Furthermore, the colorectal cancer (CRC) model was medium constrained to predict metabolite exchanges between the tumour populations.

### 2.1. Materials

Single-cell and bulk data from the matched context were downloaded from Gene Expression Omnibus (GEO) [31], Genotype-Tissue Expression (GTEX) [32], The Cancer Genome Atlas (TCGA) [33], and EBI Expression Atlas [34] (Supplementary Table S1), and preprocessed with the Seurat package [28] using the workflow described in the tutorial by the Satija Lab (https://satijalab.org/, accessed on 1 October 2021). In short, the single-cell RNAseq data are natural log-transformed before reducing the dimension of the dataset. Then, cells are clustered by performing a sequence of K-nearest neighbours, Jaccard similarity and modularity optimization processes within the top range of principal components. The results are visualised in UMAPs to obtain an overview of the clustering of the cells (Supplementary File S1).

### 2.2. Equipment

scFASTCORMICS was coded in MATLAB and required the installation of the COBRA toolbox [25], rFASTCORMICS [12] and the IBM CPLEX solver https://www.ibm.com/analytics/cplex-optimizer. All experiments were run on i5 core laptops.

## 3. Procedure

### 3.1. Parameter Optimization

Models can be reconstructed by using a set of default parameters obtained after parameter optimization of all datasets in Supplementary Table S2. These parameters are 1% for the REI and 0.5% for the coverage to discretize the single-cell data. Alternatively, the parameter optimization can be rerun for a specific dataset to compute the optimal REI and coverage values.

To determine the optimal discretization parameters for scFASTCORMICS, using bulk RNAseq data of the same context, three distinct objectives (compactness, completeness and specificity) are considered in a Pareto optimization approach.

Compactness (F1). Within the FASTCORE family, the inclusion of non-core reactions is minimised. These reactions are only included in the multi-cell population model if required for flux consistency of the core reactions. The pursuit of compactness is supported by the idea that biological systems are tuned by natural selection to avoid wasting energy and resources on the production of unnecessary enzymes and transporters, thus favouring compact systems.

Completeness (F2) and Specificity (F3). The single-cell expression data is compared to bulk RNAseq data of the same context mainly to consider the number of expressed genes that were not captured in the single-cell data due to noise, drop-outs or the choice of stringent thresholds. The rationale is that reactions supported by genes which are robustly expressed (>90% of the samples) in bulk data should be contained in at least one single-cell cluster model (F2) and reactions supported by genes that are not found in more than 90% the bulk data samples should not be contained in the multi-cell population model (in of the cluster sub-models) (F3). The bulk RNAseq data is thereby discretized into expressed, unknown expression and not expressed as previously described [12], allowing the definition of the core reactions for the bulk dataset.

A Pareto optimization approach was used to determine the parameter setting (REI, coverage) that produces models with the FASTCORE approach best fulfilling these three objectives using the following formula:

Find (REI, coverage) that:

(1) $$\mathrm{Minimize}\;F=\frac{F1+F2+F3}{3},$$

(2) $$F1=\frac{\left|A\right|-\left|C\right|}{\left|A\right|},$$

where |*A*| and |*C*| are the number of reactions in the multi-cell population model and the core set (union of all core reactions of all clusters), respectively. *F*1 represents the fraction of the reactions in the multi-cell population model that is not supported by the single-cell data.

(3) $$\;F2=\frac{\left|BE\;\cap \;N\right|-\left|BE\cap A\right|}{\left|BE\cap N\right|},$$

where *BE* and *N* stand for expressed reactions according to the bulk data and the reactions included in the generic input model, respectively. *F*2 captures the rate of metabolic reactions that are under the control of expressed genes according to the bulk data that failed to be included in the multi-cell population model.

(4) $$F\;3\;=\frac{\left|BU\cap A\right|}{\left|BU\cap N\right|},$$

where *BU* represents the reactions in the generic metabolic reconstruction deemed as not active by the bulk data. *F*3 captures the rate of reactions which had to be included in the multi-cell population model, despite not being expressed according to the bulk data, due to consistency constraints. This aim of obtaining a high specificity is equivalently formulated here as aiming for a low False Positive Rate (FPR).

In the Pareto optimization, F is minimised to determine the optimal values for the parameters REI and coverage, which allows reconstruction of a compact network with a maximum of metabolic reactions tagged as active and a minimum of reactions identified as inactive in the context of interest, both according to the considered bulk data.

Practically, each combination of coverage and REI settings between 5 and 100% was tested in steps of 10% for values above 10%. As the optimal coverage value was at the lower bound, values between 0.25% and 1% were also tested. For each cluster, genes with expression above the REI threshold were considered expressed, and if the number of cells that expressed these genes was higher than the coverage threshold, the discretization score of that gene was set to 1, otherwise the score equaled 0. The obtained scores were then mapped to the expanded model according to the Gene Protein Reaction rules (GPR rules) using the map_expression_2_data function of rFASTCORMICS [12].

### 3.2. Quality Check

While the parameter optimization was run on all datasets in Supplementary Tables S1 and S2, the data analysis is exemplified in this paper for Datasets 1 and 2 only. Dataset 1 contains 5 clusters: 3 epithelial cell clusters, a T and a B cell cluster and Dataset 2 contains 3 normal mucosa (NM) epithelial clusters and a B cell cluster. The Jaccard similarity index (JSI) of each couple of cluster sub-models (*CSM*) was computed by dividing the number of shared reactions by the total number of reactions.

(5) $$\mathrm{JSI}=\frac{\left|CSMi\;\cap CSMj\right|}{\left|CSMi\;U\;CSMj\right|}.$$

The models were then clustered according to this similarity index. Lists of genes specific to each cluster (or set of clusters corresponding to the same cell type) were uploaded to EnrichR [35] to verify if the ontology terms retrieved from EnrichR match the cell identity found with the Seurat package. A pathway analysis was further performed by computing the presence rate of each pathway. In the Recon 3D model, each reaction is assigned to a unique metabolic pathway called subsystem. For each subsystem, the number of reactions present in the multi-cell population model is divided by the number of reactions present in the generic input model to obtain a presence rate. For representation reasons, only pathways with at least 5 reactions and a difference of 0.1 in mean presence rate between the CRC epithelial and CRC immune clusters are shown.

### 3.3. Metabolite Exchange Prediction

Optionally medium constraints can be applied during the reconstruction. In short, the input reactions of the expanded input model for organic metabolites that are absent from the medium are closed. FASTCC is run to eliminate reactions that were blocked due to this constraint. A new expanded input model is created, and the CRC multi-cell population is reconstructed as explained above.

For the CRC multi-cell population model, the biomass reactions were set as objective functions. The coefficients for the optimization were weighted according to the fraction of cells contained in each cluster. Accordingly, the sum of the coefficients is equal to 1. To ensure a non-zero minimal flux in each cluster, the lower bounds of each biomass reaction was set to 10% of the predicted maximal flux for each biomass function when set as sole objective. A Flux Variability Analysis (FVA) was run using the fluxVariabilty function of the COBRA toolbox to determine the possible flux range for each reaction required to maximize the combined objective. For each cluster, the reactions that exclusively import or export reactions a metabolite between the cytoplasm and the microenvironment were identified and summed up to obtain a minimal and maximal import/export value. These maximal and minimal import values were averaged. For representation reasons, reactions only metabolites that had a predicted absolute max flux value above 100 for one of the clusters are depicted.

## 4. Results

### 4.1. scFASTCORMICS Allows Building Compact, Complete and Specific Models Based on Single-Cell RNAseq Data

Metabolic multi-cell population models will allow exploring the metabolism and metabolic interactions of different types of cells at a higher resolution than the models based on bulk RNAseq data. To this aim, we developed scFASTCORMICS, a novel workflow based on single-cell RNAseq data which generates genome-scale metabolic network models of the identified cell populations, as well as of the metabolite exchanges between them (Figure 1). This is mimicking the metabolism and metabolic interactions of different cell populations in a tissue, organ or tumour. Technically, a core expansion approach is used based on a core set of reactions which are obtained from the discretization of the single-cell RNAseq data and a generic metabolic reconstruction. To reduce the uncertainty from the high noise level and drop-out rates of the single-cell RNAseq data, reference-matched bulk RNAseq data can be used to obtain the single-cell discretization parameters by optimising for compactness, completeness and specificity (see Methods for a detailed description of the workflow).

Context-specific metabolic models reconstructed from bulk RNAseq data typically include between 3000 and 5000 reactions depending on the modelled tissue and the used generic input reconstruction [12]. However, the sizes of the multi-cell population models built with scFASTCORMICS are much larger. Depending on the number of modelled clusters, i.e., the number of included cell populations, the expanded input models (Figure 1) ranged from 39,280 to 156,800 reactions and the multi-cell population model from 17,429 to 80,161 reactions (Supplementary Table S2). Thus, more detailed descriptions of population-specific metabolic pathway patterns are obtained by these models compared to bulk RNAseq-based models with averaged pathway patterns.

**Figure 2:**
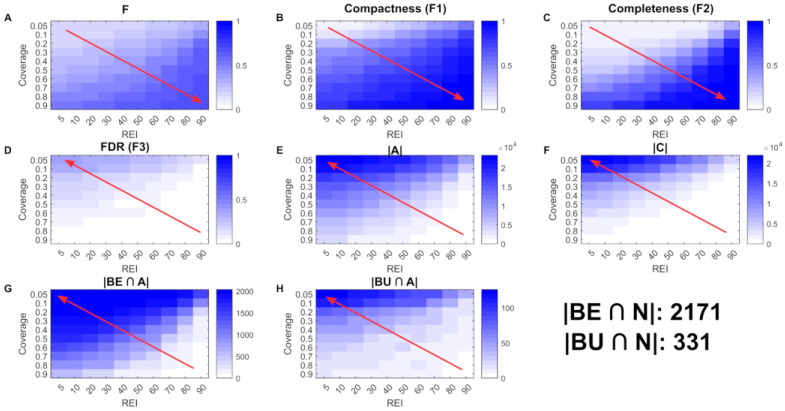
Parameter optimization. A Pareto optimization (F score, subfigure (**A**)) was performed to determine the parameter setting (Ranked Expression Intensity and coverage) that conciliates the 3 objectives: compactness (F1, subfigure (**B**)), completeness (F2, subfigure (**C**)) and specificity (F3, subfigure (**D**)). The heat maps display the scores for F, F1, F2, and F3, the number of reactions in the multi-cell population model (A, subfigure (**E**)) and core reactions (C, subfigure (**F**)), as well as the number of reactions included in the multi-cell population model identified to be always active (BE, subfigure (**G**)) and inactive by the bulk data (BU, subfigure (**H**)). The lighter the colour, the lower the F, F1, F2, F3 and the other factors used in the Pareto optimization. The red arrows indicate the way in which the F scores increase with the different parameter settings of coverage and REI.

As mentioned above, for the reconstruction of multi-cell population models, scFASTCORMICS follows three objectives that need to be balanced: compactness, completeness and specificity (equivalent to a low FPR). Within scFASTCORMICS, a core set of reactions is defined based on the single-cell data, which is expanded to obtain a compact consistent model by adding a close-to-minimal set of non-core reactions. The number of core and non-core reactions depends on two parameters: REI and coverage (see Methods). To find the optimal parameter setting for REI and coverage, hundreds of multi-cell population models were reconstructed with various REI and coverage values. The models were then assessed according to their compactness, completeness and specificity by applying a Pareto optimisation approach (see Procedure ). The heat maps (Figure 2) represent the different factors of the Pareto formula for the chosen example Dataset 1. Scores for the overall objective to be minimised (F) and the different factors of the equation are shown for the set of examined coverage and REI values. The overall score F (Figure 2A) is increasing with the REI and coverage (from lower scores in the top left and higher scores in the bottom right, as indicated by the red arrow). The sub-scores for compactness (F1) (Figure 2B) and completeness (F2) (Figure 2C) show the highest scores for the highest REI and coverage values (lower right corner). In terms of the core reaction set, we can observe that the higher the two parameters (coverage and REI), the smaller the core reaction set (Figure 2F), and hence, more non-core reactions are added to the multi-cell population models. Hence, the compactness objective (F1) tends to favour low coverage and REI. The same is true for completeness (F2). High REI and coverage values reduce the number of reactions supported by the bulk RNAseq data, while the multi-cell population model (A) (Figure 2E) follows the same trend as C (Figure 2F). The FPR (F3) (Figure 2D) heat map displays the inverse behaviour to the two previous factors, with a high score region in the upper left corner, suggesting that a large fraction of reactions unsupported by the bulk data is included in the model for lower thresholds. The Pareto compromise of the 3 objectives results in a zone with low overall scores in F in the top left corner. BE follows the same trend as F1 and F2. |BE ∩ A| (Figure 2G) is the number of bulk RNAseq-supported reactions that are present in the input model and were included in the multi-cell population model, whereas |BU ∩ A| (Figure 2H), shows the same behaviour as F3. To verify the robustness of the obtained optimal parameter setting, the workflow was run on 20 different datasets, mainly from colon cancer cells, pancreatic islets and healthy control mucosal samples (Supplementary Table S1). All data sets displayed similar heat maps to the example shown in Figure 2 and the REI and coverage values ranged between 1 and 20 and 0.25 and 5%, respectively (Supplementary Table S2). The default discretization parameter setting was set to the median of these optimal parameters, resulting in REI = 1% and coverage = 0.5%. The F-scores obtained with optimized REI and coverage varied between 0.1072 and 0.207. Reconstructing the model with default REI and coverage did not increase the F-score by more than 10% for 15 out of 20 datasets. However, for the 5 remaining datasets, the increase was between 11 and 30%. The running time for scFASTCORMICS without parameter optimization is about ten minutes to two days and depends on the number of clusters, as the size of the stoichiometric matrix of the expanded model increases rapidly with the number of clusters. With parameter optimization, the running time ranged from a few hours to six days for the different tested datasets on an i5 core laptop.

### 4.2. The Multi-Cell Population Model Captures Metabolic Variation among Cell Populations

**Figure 3:**
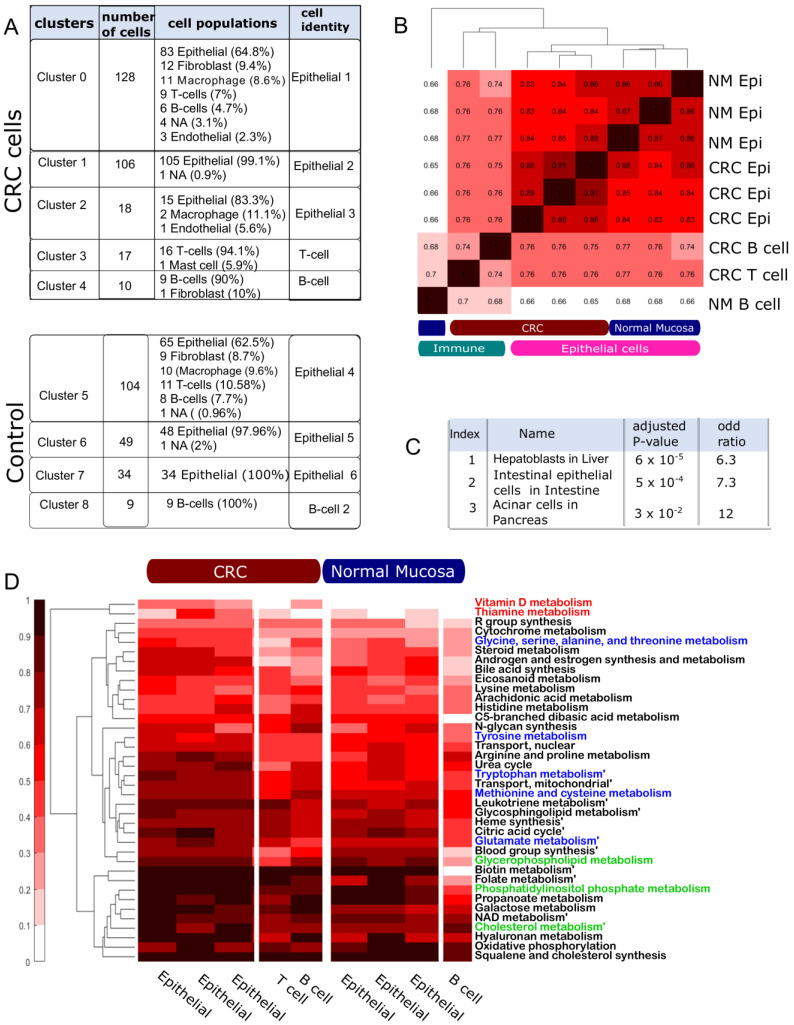
Pathway analysis: scFASTCORMICS captures metabolic variations between the cell populations for a colorectal cancer (CRC) example (Datasets 1 and 2): (**A**) Composition of the 5 cell clusters in Dataset 1 and 4 clusters in Dataset 2 identified by the pre-processing of the single-cell data by Seurat. (**B**) Jaccard similarity index of the different cluster sub-models in the CRC and normal mucosa multi-cell models. The darker the colour, the higher the similarity level. (**C**) Enrichment analysis of the epithelial-specific genes included in the multi-cell population model. An enrichment was found in the epithelial clusters for Intestinal epithelial genes in the Descartes cell types and tissues library. (**D**) Pathway analysis representing the presence rate of Recon3D pathways with more than 5 reactions and a mean differential presence rate above 0.1. Pathways related to biosynthesis, amino acid metabolism, redox balance and inflammation are represented in green, blue, red, and purple, respectively.

To exemplify the relevance of scFASTCORMICS for the study of cancer metabolism, we focused more intensively on one example (Datasets 1 and 2) on the metabolism of colon cancer tumours versus control (GSE81861) (Supplementary Figure S1). Two multi-cell population models containing 5 clusters were reconstructed using scFASTCORMICS (Figure 3A). First was a CRC model that included 3 epithelial subpopulations (epithelial 1–3), and T and B cells subpopulations. The second model corresponded to normal mucosa (NM) and was composed of epithelial 4–6 and B cells 2. The number of reactions in the CRC expanded input model and the CRC multi-cell population model was 48,320 and 22,961, respectively (Table 1). The NM expanded input model and the NM multi-cell population model were compacter as the dataset only contained 4 clusters against 5 for the CRC model. In addition, the NM sub-models were slightly smaller than the CRC sub-models (mean CRC epithelial sub-model size: 4650 vs 4303 for the NM epithelial and 3988 vs 3457 for the B cell sub-models). Further, the sub-models corresponding to the immune cells included fewer reactions than the epithelial clusters. To assess if the scFASTCORMICS model was specific enough to capture metabolic variations between the different clusters, the Jaccard Similarity Index (JSI) was computed for each pair of clusters in the model (Figure 3B). For the cancer models, the epithelial cells had the highest number of shared reactions, with JSI between 0.88 and 0.91 indicating also some metabolic variation between the three epithelial clusters. B (cluster 4) and T cells (cluster 3) in the cancer samples only had a similarity rate of 0.74 to each other and between 0.75 and 0.76 for the epithelial cells (clusters 0–2). The epithelial cells in the normal mucosa clustered together (JSI: 0.86–0.87). NM and CRC epithelial subclusters had a higher similarity (0.83–0.88). They formed, however, two separate clusters. In addition, the immune sub-models cluster together. However, a higher similarity could be observed between the immune cells in the CRC environment vs NM (0.68–0.7). The lower JSI of NM sub-models can partially be explained by their smaller size, which confers more weight to every variation. However, it also indicates that the added reactions in the cancer multi-cell population model tend to be present between the 3 epithelial clusters suggesting the existence of common rewiring strategies.

The term “Intestinal epithelial cells in the intestine” was among the top enriched ontology terms for the epithelial-specific genes according to the Descartes cell types and tissues library of the EnrichR web tool, with an adjusted p-value of 5 × 10^4^ (Figure 3C). Further among the pathways with the highest increase in reaction presence rate between the epithelial and the immune cells and between CRC and NM more generally are biosynthetic pathways (glycerophospholipids, cholesterol, squalene and cholesterol synthesis and phosphatidylinositol phosphate, Figure 3D in green), amino acid metabolism (Figure 3D in blue), pathways implicated in inflammation (eicosanoid, leukotriene and arachidonic acid metabolism Figure 3D in purple) and redox balance (vitamin D, folate and thiamine, Figure 3D in red), which is consistent with the literature on pathways commonly upregulated in cancer cells (see Discussion). Other pathways such as propanoate metabolism, galactose, and hyaluronan metabolism, often described as potential markers or targets in CRC (see Discussion), were predicted to have a higher presence rate in the CRC epithelial sub-models. scFASTCORMICS captures metabolic variations that are consistent with the literature.

**Figure 4:**
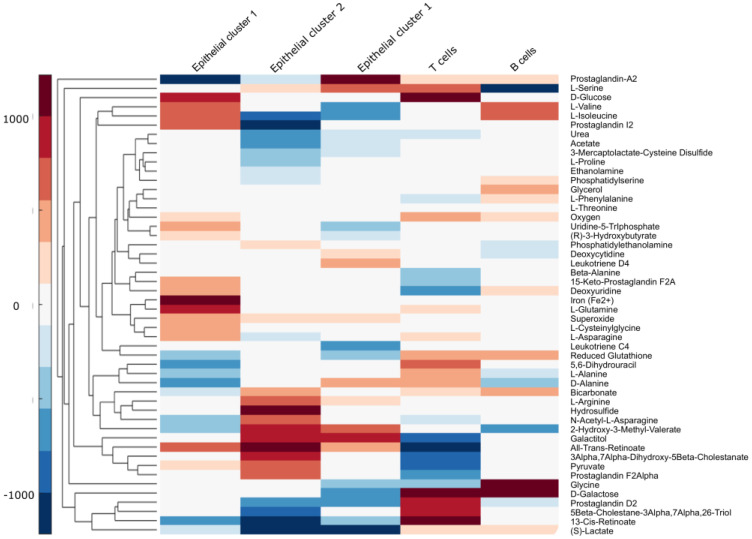
Metabolic exchanges: A Flux Variability Analysis was run while optimizing for the biomass reactions of the 5 clusters. The optimization coefficients were weighted according to the population size. For each biomass function, a minimal flux of 10% of the flux obtained when the biomass is unique optimization function was imposed. The cluster gram depicts the exchange between the cytoplasm of the sub-models and the microenvironments. As metabolites can participate in several transport reactions, the average fluxes were summed up. The exports and imports are depicted in red and blue, respectively.

Further, to predict metabolic exchanges between the subpopulations of the CRC multi-cell population model (Figure 4), a flux variability analysis was performed using the biomasses of the 5 sub-models as objective functions (see Procedure). Notably, lactate is predicted to be secreted by the epithelial clusters and to be uptaken mainly by B cells but also by T cells. According to the FVA analysis, T cells prefer glucose as the main carbon source and hence are competing with the epithelial clusters (mainly Epithelial 1) for this carbon source, while B cells and to some extent T cells complement their energy source with glutamine a coveted carbon and nitrogen donor. Tumour cells also secrete prostaglandins, notably prostaglandins D and 13-cis-Retinoic acid (a type of Vitamin A) that are uptaken by T cells. Both were described to have an immunomodulation effect on immune cells [36,37]. Galactose, a known biomarker [38] of CRC is also predicted to be exchanged between the CRC epithelial cell populations and the immune cells. Amino acids play a central role in the metabolism of cancer cells but are also required for the effector functions of immune cells forcing immune cells to compete for some amino acids while others are exchanged between the immune cells and the CRC epithelium. Two main mechanisms govern the interaction between the epithelial cells and immune cells: (1) a completion for carbon and nitrogen sources and (2) the secretion of metabolites by the CRC epithelial cells that reduce the effector cells in the microenvironment.

## 5. Discussion

scFASTCORMICS allows building metabolic multi-cell population models from single-cell RNAseq data. The workflow requires as inputs a generic metabolic reconstruction and single-cell expression data clustered according to their expression profiles by Seurat or any other clustering tool. The data can be discretized using the default parameter setting (REI: 1 and coverage 0.5%) to obtain a set of core reactions to which a close-to-minimal set of non-core reactions is added. The default parameter settings were obtained with Recon3D, so the workflow might need to be rerun for other generic reconstructions. Alternatively, Pareto parameter optimisation can be run to tailor the parameters to the single-cell dataset using matched-reference bulk RNAseq data as additional input. For Recon3D, the optimal parameter setting varied slightly from one dataset to another, but overall, the default parameter settings differed only by 10% from the optimal parameter setting for most datasets. However, for 5 out of 20 datasets, the increase was between 10 and 31%, notably 31% for Dataset 1. scFASTCORMICS could theoretically be used to build an integrated single-cell-based context-specific model (single-cell model) in which every subnetwork represents an individual cell instead of a cell population as we showed here. However, with the large number of cells in single-cell RNAseq datasets the size of the stoichiometric matrix might rapidly exceed the memory capacity of most computers and challenge most solvers. Here, for the multi-cell population models, the size of the expanded input models and the reconstructed context-specific models were largely above 100,000 reactions, with a maximum number of around 156,800 reactions for a 17-cluster model. For these model sizes, the used IBM CPLEX solver could produce consistent network models as performed with FASTCC [22], but additional tests might be required to determine the limits of other solvers. Furthermore, the computational cost associated with downstream analysis such as random sampling or multiple gene and reaction knock-outs might make the analysis practically infeasible or with restricted utility for single-cell models. Further developments in theory and computation are needed here to overcome these limits. Meanwhile, the building and analysis of multi-cell population models while increasing the computational cost will probably remain feasible on ordinary laptops and computers.

The models reconstructed with scFASTCORMICS can be used as input for flux balance analysis, flux variability analysis, random sampling, gene or reaction deletions or any downstream analysis commonly performed on context-specific metabolic models. However, the computational times might be longer due to the larger size of the networks. Further, for analysis requiring the definition of an objective function, the maximization of an objective function per population might be necessary. In this paper, we run FVA while optimizing simultaneously for all biomass functions. The optimization coefficients were weighted in function of the population sizes. To avoid the weight of a larger cluster preventing the other biomass functions to carry a flux, we set the lower bound of the biomass functions to 10% of the value obtained for each biomass when the latter is the sole objective; 10% is an arbitrary threshold. Other values would need to be tested to explore the robustness of the prediction on various datasets and determine the optimal weights to assign to the cluster. More advanced approaches such as a multi-objective formulation of FBA could have been employed to obtain a better approximation of the Pareto frontier [39], but a more through benchmark would be required to assess its performance with models with over 20,000 reactions.

The preliminary results on the CRC Datasets 1 and 2 (GSE81861) showed that scFASTCORMICS can capture variations that are consistent with the cell annotations found by Seurat. Furthermore, pathway analysis showed the activation of biosynthetic pathways, and amino acid metabolisms required to sustain high proliferation rates as well as vitamins and more generally pathways implicated in the redox maintenance in the CRC epithelial clusters. This is consistent with cancer cells undergoing the Warburg effect. The Warburg effect is a metabolic rewiring of the metabolism of cancer cells to produce energy preferably through anaerobic glycolysis even in presence of oxygen. This allows using the intermediates from the tricarboxylic acid (TCA) for nucleotide, amino acid, and lipid biosynthesis [40]. The biosynthesis of amino acids was described to be upregulated in many cancers. Amino acids, found to be upregulated in our analysis, are required for biosynthesis pathways notably of proteins but are also precursors of nucleotides. They can also be converted to glucose or α-keto acid for energy production. In addition, they allow redox maintenance through the synthesis of glutathione, from glutamate, cysteine, and glycine [41]. Cancer cells, due to the accelerated metabolism, tend to produce higher rates of ROS. While lower concentrations of ROS favour tumorigenesis, higher rates induce apoptosis [42,43]. The upregulation of glycolysis further allows the fuelling of the pentose phosphate pathway and the reduction of NADPH from NADP+ required for replenishing reduced glutathione, a potent antioxidant [44,45]. Other pathways that contribute to regulating ROS such as vitamins tend to have a higher presence rate in the CRC epithelial clusters, notably thiamine, folates and vitamins. Thiamine acts as an antioxidant and facilitates the use of carbohydrates for energy production [46], while vitamin D was shown to reduce inflammation and oxidative stress [47]. Arachidonic acid, eicosanoids such as prostaglandins, and leukotrienes play an essential role in inflammation, regulation of inflammation and cytokines production [46]. Other pathways found by our analysis were shown to be disrupted in CRC, notably Galactose, and Propanoate pathways, and Hyaluronic metabolism. Galactose was found in the stool of CRC patients and the galactose metabolism was shown to be altered in CRC compared to adjacent mucosa [47]. Galactose was further shown to force T cells to switch from glycolysis to OXPHOS in vitro, which could reduce their effector function and potentially explain the exchange of galactose between CRC epithelial cells and immune cells predicted in our model [38]. Concerning propanoate metabolism, an up-regulation of this pathway reduces the availability of short-chain fatty acids mainly produced by the microbiota. Organic acids, among others, induce apoptosis of cancer cells [48]. Finally, hyaluronic acids are unbranched heteropolysaccharides that participate in the extracellular matrix and are secreted in the TME of cancer cells and were shown to play a role in stroma homeostasis maintenance, immune escape, stemness, migration, proliferation and invasion [49]. Hyaluronic acids also form a physical barrier that reduces immune cell infiltration in the TME [50]. Perturbations in hyaluronic metabolism cause a remodelling of the extracellular matrix that favours angiogenesis, CAF and macrophage recruitment [51].

We also predicted metabolite exchanges between the different clusters in the CRC sample. To this end, we assigned the optimization coefficients according to the number of cells in the clusters. Our predictions showed that T cells rely on glucose but also uptake lactate secreted by the CRC epithelial cells. Activated T cells that rely on glycolysis for energy production compete for glucose with tumour cells that underwent the Warburg effect. The deprivation of glucose in the microenvironment can cause T cell inactivation which cannot fulfil their effective functions anymore [38]. The Warburg effect cause CRC epithelial cells to secrete lactate in the microenvironment that, according to our prediction, is mainly taken up by B cells and, to some extent, T cells. The accumulation of lactate causes acidosis of the TME which hinders immune cell activation and induces immune suppression [38]. The metabolization of lactate by T cells was shown to reduce NAD+ to NADH. The depletion of NAD+ blocks 3-phosphate dehydrogenase (GAPDH) and 3-phosphoglycerate dehydrogenase, two enzymes required for the synthesis of serine from glucose, a metabolite required for T cell proliferation [48]. Activated B cells are not as dependent as T cells on glucose as they can use oxidative phosphorylation as well as glycolysis for energy production. Notably, the effect of lactate on B cells remains unclear [49]. The model predicts an uptake of serine by T cells which is coherent with a compensation for the blockage of the serine biosynthesis from glucose. In addition, glucose and lactate, T, B and CRC cells can uptake glutamine to fuel their metabolism. Glutamine is a source of carbon and nitrogen for anabolic pathways in tumours but also T cells that require glutamine for their effector functions, i.e., production of cytokines [50].

Besides lactate, cancer cells are also predicted to release prostaglandin D in the TME, which is consistent with the Phospholipase D2 (PLD2) overexpression and secretion by CRC cells [51]. Prostaglandin D2 suppress acute immune response [36]. Another immune cell modulator 13-cis-Retinoic acid was also predicted to be secreted by CRC epithelial cells and to be taken up by T cells [52]. Retinoic acid is a known T-cell modulator [37]. Finally, amino acids are key players in the metabolism of cancer cells but are required for the effector functions of immune cells. While some amino acids can be obtained by the conversion of glucose or glutamine in tumour cells, others have to be imported from exogenous sources. Notably, CRC tumours lose the ability to synthesise arginine that has to be uptaken from the TME [53]. The upregulation of biosynthetic pathways, glycolysis and glutamine metabolism in cancer cells but also immune cells favour the release of amino acids and pyruvate into the TME, explaining at least partially some of the exchanges.

More generally, the predictions are consistent with the literature; however, a survey of a larger number of more recent datasets with a larger number of cells and patients such as [54,55,56] would be required to explore more deeply the metabolism of the colorectal cancer cell populations and confirm our preliminary results.

## 6. Conclusions

scFASTCORMICS builds multi-cell population models that capture metabolic variations between these populations. The discretization approach used by scFASTCORMICS is robust and hence parameter optimisation based on reference bulk data is not required for every dataset. As shown for the colorectal cancer example, the scFASTCORMICS model can provide insights into metabolic differences between tumour epithelial cells and infiltrated immune cells. This novel workflow might become the computational backbone for many metabolism research projects involving single-cell expression data.

## Figures and Tables

**Table 1:** Summary of the different models for colorectal cancer (CRC) and normal mucosa (NM) examples (Datasets 1 and 2). The number of reactions, metabolites and genes are given for the input generic model, expanded input models, multi-cell population models and all the sub-models. The multi-cell models include, besides the reactions, metabolites and genes present in all sub-models, transport and exchange reactions and metabolites and their associated genes.

Models	Number of Reactions	Number of Metabolites	Number of Genes
Input generic model (Recon 3D)	10,600	5835	1883
Expanded input model CRC	48,320	24,495	11,240
Expanded input model NM	39,280	20,220	8992
Multi-cell population CRC	22,961	16,292	9231
Multi-cell population NM	17,429	12,758	7327
Cluster 0 sub-model (CRC epithelium)	4731	3165	1896
Cluster 1 sub-model (CRC epithelium)	4678	3157	1951
Cluster 2 sub-model (CRC epithelium)	4542	3078	1844
Cluster 3 sub-model (CRC T cell)	3902	2777	1777
Cluster 4 sub-model (CRC B cell)	3988	2835	1763
Cluster 5 sub-model (NM epithelium)	4224	2993	1841
Cluster 6 sub-model (NM epithelium)	4344	3032	1916
Cluster 7 sub-model (NM epithelium)	4340	3012	1871
Cluster 8 sub-model (NM B-cell)	3457	2513	1699

## Data Availability

The original code of scFASTCORMICS, is available on Zenodo: 10.5281/zenodo.7307915. The input data are stored: 10.5281/zenodo.7294889, 10.5281/zenodo.7294895, 10.5281/zenodo.7294902, 10.5281/zenodo.7294911, 10.5281/zenodo.7294920, 10.5281/zenodo.7294926, 10.5281/zenodo.7294932, 10.5281/zenodo.7294936, 10.5281/zenodo.7294938 10.5281/zenodo.7294947, 10.5281/zenodo.7294953, 10.5281/zenodo.7294960, 10.5281/zenodo.7294968, 10.5281/zenodo.7294974, 10.5281/zenodo.7294984, 10.5281/zenodo.7295001, 10.5281/zenodo.7295029, 10.5281/zenodo.7295053, 10.5281/zenodo.7295079, 10.5281/zenodo.7295091, 10.5281/zenodo.7295118, 10.5281/zenodo.7295505.

## Supplementary Materials

The following supporting information can be downloaded at: https://www.mdpi.com/article/10.3390/metabo12121211/s1. Table S1: Datasets used for the parameter optimisation. Table S2: The variance of the REI and coverage. Figure S1: Quality Control: UMAP plot of the single-cell RNAseq data of dataset1 obtained after running the Seurat pipeline
