# Supplementary material for: scFASTCORMICS: A Contextualization Algorithm to Reconstruct Metabolic Multi-Cell Population Models from Single-Cell RNAseq Data
Source: Metabolites. 2022 Dec 2;12(12):1211. doi: 10.3390/metabo12121211 (PMC9785421; doi:10.3390/metabo12121211)
Supplement: Supplementary file 1 [file metabolites-12-01211-s001.zip › metabolites-1955477-SI.pdf]

scFASTCORMICS: A contextualization algorithm to reconstruct metabolic multi-cell population models from single-cell RNAseq data- Supplementary File

Maria Pires Pacheco, Jimmy Ji,Tessy Prohaska, María Moscardó García and Thomas Sauter

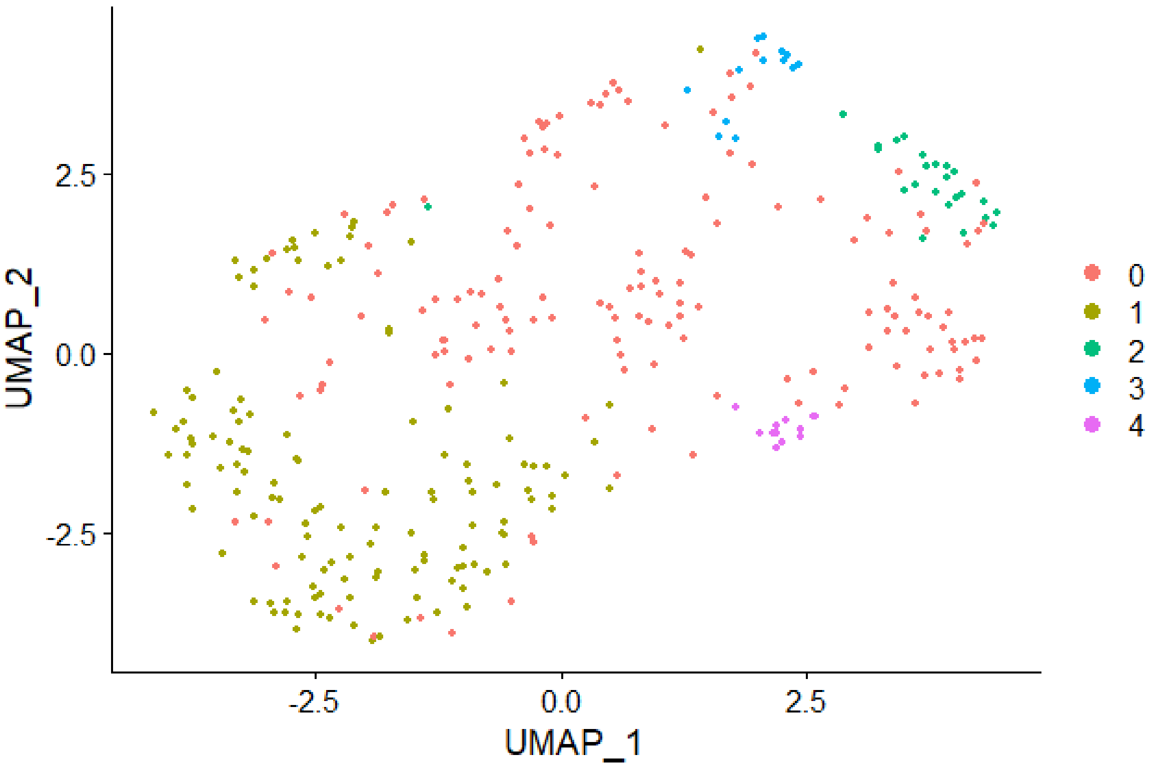

Figure S1: Quality Control: UMAP plot of the single-cell RNAseq data of dataset1 obtained after running the Seurat pipeline

Table S1: **Datasets used for the parameter optimisation**. The accession numbers of the single-cell RNAseq and the matching bulk RNAseq data as well as the tissue and cell type and the number of samples/cells are given for the 20 datasets.

Tissue/Cell type	Single-cell RNAseq data accession number	Number of cells	Bulk RNAseq accession number	Number of samples
Colorectal cancer (CRC)				
CRC tumour all cells	GSE81861	279	Lee *et al*(2020)	26
CRC normal mucosa All cells	GSE81861	202	Healthy colon from GTEX	376
Human islet single-cells (HISC)				
Pancreas donor 1	GSE114297	1551	Pancreatic islet cell from EBI Expression Atlas	121
Pancreas donor 2		918		
Pancreas donor 3		1954		
Pancreas donor 4		1503		
Pancreas donor 5		1052		
Pancreas donor 6		404		
Pancreas donor 7		1860		
Pancreas donor 8		1557		
Pancreas donor 9		2250		
Pancreas donor 10		2322		
Pancreas donor 11		2141		
Pancreas donor 12		2786		
Immune cells in pancreatic carcinoma (PACA)				
CD8 T-cells in PACA	GSE156728	5886	GSE156278	8
CD4 T-cells in PACA		3854		
Metastatic Merkel cell polyomavirus Merkel cell carcinoma (MCC)				
PBMC metastatic MCC	GSE117988	11327	PBMC from EBI Expression Atlas	7
Liver cancer cell				
Liver cancer set 1	GSE125449	5058	Liver cancer from TCGA (GSE62944)	377
Liver cancer set 2		4801		
Breast cancer cell				
Breast cancer set	GSE180286	3123	Breast cancer from TCGA (GSE62944)	1122

Table S2: **The variance of the REI and coverage**. The optimal REI and coverage, as well as the F score obtained with default and optimal REI and coverage were computed across 20 single-cell RNAseq datasets with different numbers of clusters and cell and tissue types to assess their variance. To evaluate the computational demands of the parameter optimization (PO), the size of the expanded and multi-cell population (MCP) models, the number of clusters and the running time with and without the PO is also given.

Tissue/Cell type	Number of clusters	REI (in %)	Coverage (in %)	Optimal F score	Default F score	Expanded model number of reactions	MCP model number of reactions	Running time (with PO (hours))	Running time (without PO (hours))
Colorectal cancer (CRC)									
CRC tumour all cells	5	20	2	0.167	0.217	48320	22961	6	0.35
CRC normal mucosa All cells	4	20	5	0.109	0.130	39280	17429	5	0.2
Human islet single-cells (HISC)									
Pancreas donor 1	10	1	0.75	0.166	0.182	93520	43078	90	4
Pancreas donor 2	7	1	0.25	0.169	0.172	66400	30877	15	1.5
Pancreas donor 3	11	5	1.25	0.148	0.152	102560	47787	60	4
Pancreas donor 4	8	5	0.25	0.160	0.161	75440	36277	24	2
Pancreas donor 5	6	1	0.5	0.139	0.139	57360	27545	12	1
Pancreas donor 6	4	1	0.25	0.142	0.142	39280	17618	3	0.28
Pancreas donor 7	8	1	1	0.148	0.158	75440	36202	27	3
Pancreas donor 8	9	1	0.25	0.174	0.179	84480	40790	84	3
Pancreas donor 9	11	1	0.75	0.153	0.157	102560	48930	46	5
Pancreas donor 10	10	1	0.5	0.165	0.165	93520	44594	37	5
Pancreas donor 11	10	1	0.5	0.174	0.174	93520	42657	33	4
Pancreas donor 12	10	1	0.25	0.182	0.183	93520	43563	37	5.5
Immune cells in pancreatic carcinoma (PACA)									
CD8 T-cells in PACA	16	5	0.25	0.120	0.125	147760	68378	114	22
CD4 T-cells in PACA	10	1	0.25	0.127	0.130	93520	44108	43	8
PBMC metastatic MCC	13	1	0.25	0.187	0.208	120640	49865	77	44
Liver cancer set 1	17	10	0.25	0.200	0.231	156800	80161	137	20.5
Liver cancer set 2	17	1	0.5	0.207	0.207	156800	75405	125	20.5
Breast cancer cell									
Breast cancer set	7	5	0.25	0.107	0.126	66400	29482	18	5
